# Characterization of the Class I MHC Peptidome Resulting From DNCB Exposure of HaCaT Cells

**DOI:** 10.1093/toxsci/kfaa184

**Published:** 2020-12-29

**Authors:** Alistair Bailey, Ben Nicholas, Rachel Darley, Erika Parkinson, Ying Teo, Maja Aleksic, Gavin Maxwell, Tim Elliott, Michael Ardern-Jones, Paul Skipp

**Affiliations:** 1 Centre for Proteomic Research, Biological Sciences and Institute for Life Sciences, University of Southampton, Southampton SO17 1BJ, UK; 2 Centre for Cancer Immunology and Institute for Life Sciences, Faculty of Medicine, University of Southampton, Southampton SO16 6YD, UK; 3 Clinical and Experimental Sciences, Sir Henry Wellcome Laboratories, Faculty of Medicine, University of Southampton, Southampton SO16 6YD, UK; 4 Safety & Environmental Assurance Centre, Unilever, Colworth Science Park, Sharnbrook MK44 1LQ, UK

**Keywords:** HLA, peptidome, DNCB, keratinocyte, HaCaT

## Abstract

Skin sensitization following the covalent modification of proteins by low molecular weight chemicals (haptenation) is mediated by cytotoxic T lymphocyte (CTL) recognition of human leukocyte antigen (HLA) molecules presented on the surface of almost all nucleated cells. There exist 3 nonmutually exclusive hypotheses for how haptens mediate CTL recognition: direct stimulation by haptenated peptides, hapten modification of HLA leading to an altered HLA-peptide repertoire, or a hapten altered proteome leading to an altered HLA-peptide repertoire. To shed light on the mechanism underpinning skin sensitization, we set out to utilize proteomic analysis of keratinocyte presented antigens following exposure to 2,4-dinitrochlorobenzene (DNCB). We show that the following DNCB exposure, cultured keratinocytes present cysteine haptenated (dinitrophenylated) peptides in multiple HLA molecules. In addition, we find that one of the DNCB modified peptides derives from the active site of cytosolic glutathione-S transferase-ω. These results support the current view that a key mechanism of skin sensitization is stimulation of CTLs by haptenated peptides. Data are available via ProteomeXchange with identifier PXD021373.

Skin sensitization by haptens (small molecules of molecular mass <1000 Da capable of conjugating proteins or other larger molecules (Lepoittevin, 2006) is a form of chemical allergy leading to allergic contact dermatitis (ACD) (Kimber *et al.*, 2011). The allergy is the result of a 2-phase process: Sensitization following first exposure, and second, elicitation of an immune response. Sensitization involves penetration of the hapten into the stratum corneum of the epidermis, encountering keratinocytes as a source of endogenous proteins for modification (haptenation), and resultant secretion of proinflammatory cytokines (Banerjee *et al.*, 2004; Kaplan *et al.*, 2012; Koppes *et al.*, 2017). These signals mobilize dendritic cells, primarily epidermal Langerhans cells, which internalize and process proteins, and transport them to draining lymph nodes where they prime and expand reactive cytotoxic T lymphocytes (CTLs) (Itano *et al.*, 2003; Koppes *et al.*, 2017; Weltzien *et al.*, 1996). Elicitation is then triggered following re-exposure of a sensitized individual to the chemical sensitizer and skin recruitment and activation of allergen-specific CTLs. 

2,4-Dinitrochlorobenzene (DNCB) is commonly used as a reference contact allergen as it will readily sensitize most immunocompetent people (Friedmann *et al.*, 1983). The chemical structure of DNCB makes it preferentially reactive with nucleophilic amino acids lysine and cysteine, and to a lesser extent with arginine, histidine and tyrosine, to form 2,4-dinitrophenol (DNP) adducts (Aleksic *et al.*, 2009; Landsteiner and Jacobs, 1935; Lepoittevin, 2006; Parkinson *et al.*, 2018). We have previously described DNCB haptenation of the HaCaT cell proteome following 4 h exposure (Parkinson *et al.*, 2020). DNCB penetrates skin into the epidermis and induces the migration of Langerhans cells following protein haptenation (Pickard *et al.*, 2009), modifies cellular proteins (Pickard *et al.*, 2007), and is removed from cell by forming inactive conjugates of the antioxidant glutathione (Townsend *et al.*, 2003). When glutathione becomes depleted, the remaining DNCB can haptenate protein more freely, suggesting cellular detoxification regulates immunity to DNCB (Jacquoilleot *et al.*, 2015; Pickard *et al.*, 2007).

Human peripheral blood mononuclear cells (PBMC) cultured with DNCB can prime naive CTLs (Dai and Streilein, 1998), and studies in sensitized mice and humans show that DNCB raises both CD4+ and CD8+ CTL responses, but not in nonsensitized individuals (Dearman *et al.*, 1996; Newell *et al.*, 2013; Pickard *et al.*, 2007). Use of synthetic peptides has shown that DNCB modified peptides bound to class I human leukocyte antigen molecules (HLA-I) can prime HLA-I restricted CD8+ CTL responses (Gagnon *et al.*, 2003). It has been subsequently observed that most CTLs reactive to one haptenated peptide could also react with a synthetic peptide modified with one or more of 8 different sensitizers (Gagnon *et al.*, 2006). These studies indicate individuals may contain CTL clones capable of recognizing haptenated peptides and that these clones can recognize and react to peptides haptenated at the same position by a variety of different chemicals. Recognition of the β-lactam ring of penicillins by class II HLA restricted CTLs supports the idea that direct presentation of haptenated peptides is key to β-lactam allergy (Padovan *et al.*, 1996). This is further supported by the recent identification of peptides haptenated by β-lactam antibiotic flucloxacillin and presented by class I HLA-B*57:01 (Waddington *et al.*, 2020). Moreover, evidence of class I and class II CTL recognition of haptenated peptides derived from sensitizers such as 2,4,6-trinitrochlorobenzene (TNCB) (Kohler *et al.*, 1997; Martin *et al.*, 1992, 1993a; Von Bonin *et al.*, 1992), and of glycosylated peptides (Glithero *et al.*, 1999; Speir *et al.*, 1999), suggests an important role for direct presentation of modified peptides in the CTL meditated immune response.

However, the precise mechanism of the DNCB mediated immune response in skin remains to be established. Sensitizing drugs have been shown to modify HLA-I by changing the shape and chemistry of the peptide binding groove leading to a change in the repertoire of presented peptides (the peptidome) (Illing *et al.*, 2012). This altered peptidome associated induced CTL activation and severe multisystem inflammation has been demonstrated in HLA-B*57:01 humans exposed to abacavir, and HLA-A*31:01 humans exposed to carbamazapine (Bharadwaj *et al.*, 2012). Similarly, in chronic beryllium disease the class II allotype HLA-DPB1*02:01 contains a glutamic acid at position 69 that it is suggested binds beryllium leading to an altered peptide binding conformation that is immunogenic (Dai *et al.*, 2013). It has also been shown the peptidome can be changed by other factors such as the local inflammatory milieu. For example, interferon gamma (IFN-γ) exposure can change the length distribution and peptide C-terminus biochemistry of the presented peptidome (Boulanger *et al.*, 2018; Chong *et al.*, 2018). Furthermore, as chemical sensitizers have been shown to modulate intracellular oxidative stress and the generation of reactive oxygen species (Corsini *et al.*, 2013; Esser *et al.*, 2012), and consequently modulate antigen processing (Trujillo *et al.*, 2014), it is possible that exposure to the sensitizer may modify the peptidome without direct interaction with MHC. These 3 nonmutually exclusive models provide mechanisms for how chemical sensitizers mediate CTL activation: Direct presentation of haptenated peptides, hapten modification of HLA-I creating an altered peptidome, or a hapten modulated proteome leading to an altered peptidome. The relative importance of each model is unknown. A greater understanding of the targets of haptenation and mechanisms of keratinocyte activation in the proposed OECD adverse outcome pathway for skin sensitization (Organisation for Economic Co-operation and Development, 2014) is needed for the development of risk assessments of sensitizer potency (Gilmour *et al.*, 2019; Höper *et al.*, 2017; Reynolds *et al.*, 2019). To address this, we have investigated the processing and presentation of HLA-I peptides by cells exposed to DNCB using mass spectrometry-based proteomics. We show that keratinocytes present haptenated peptides and find no evidence of other alterations to the peptidome. In addition, we find that one of the DNCB modified peptides derives from the active site of cytosolic glutathione-S transferase omega, suggesting DNCB can disrupt the machinery of glutathione-mediated detoxification (Jacquoilleot *et al.*, 2015; Townsend *et al.*, 2003).

## MATERIALS AND METHODS 

###  

####  

##### Primary human material and cell lines

An overview about the patient is given in Supplementary Table 1.

##### Cell lines and antibodies

HaCaT cells (CLS Cell Lines Service GmbH, DE) were maintained in in Dulbecco’s Modified Eagle Medium (DMEM) supplemented with 4.5 g/l glucose, 2 mM l-glutamine and 10% fetal bovine serum and 1% penicillin and streptomycin (all reagents from Gibco). Cells were maintained at 37°C and 5% CO_2_.

HLA-A*02:01 was amplified from pcDNA3 (Lewis *et al.*, 1996) using 5’ forward primer *GGATCCCCGGGTACCGCCGCCATGGCCGTCATGGCGCCC* and 3’reverse primer *CGGGGATCTGATATCATCGATTCACACTTTACAAGCTGTGAG*. Fragments were cloned into KpnI and ClaI sites within pMCFR.puromycin (Williams *et al.*, 2002) using Sequence and Ligation-Independent Cloning (Li and Elledge, 2007). All reagents sourced from Promega, United Kingdom. Correct insertion and alignment were confirmed by sequencing with internal 5’ forward *AGAGGACCTGCGCTCTTG* and 3’reverse *GGTGGCTTCATGGTCAGAGA* primers by Source Bioscience Ltd, Nottingham. HaCaT cells were transfected using FuGENE 6 (Promega) at ratio of 6:1 according to the manufacturer’s instructions. Stable transfectants were selected with puromycin (Melford Labs) at 1 μg/ml and single cell cloned to isolate a high expressing clone.

Phenotype was confirmed (Supplementary Figure 1) by staining with anti-HLA-A2 antibody BB7.2 (Parham and Brodsky, 1981) or anti-HLA-A, B, C antibody W6/32 (Barnstable *et al.*, 1978) followed by goat antimouse conjugated with FITC (Sigma-Aldrich) and analyzed by FACS (Luminex Guava easyCyte).

W6/32 monoclonal antibodies were purified from the growth medium as previously described (Parham *et al.*, 1979).

##### Human leukocyte antigen typing of HaCaT cells

An HLA typing was performed by Next Generation Sequencing by the NHS Blood and Transplant Histocompatibility and Immunogenetics Laboratory, Colindale, United Kingdom.

##### Sensitizer exposure of keratinocytes

Cells exposed to sensitizer were cultured for 24 h in serum free DMEM-F12 culture medium (Gibco) before washing and harvesting as previously described (Boukamp *et al.*, 1988). Dinitrochlorobenzene (DNCB) (99% purity; MW 202.55 Da) was obtained from Sigma, and DNCB-D_3_ (99% purity; MW 205.57 Da) was obtained from QMX Laboratories. Stock solutions of each chemical were made by dissolving a 50:50 mix, by molar concentration, of deuterated, and unlabeled chemical in ethanol to a final concentration 100 mM. DNCB stock solution was added to cells in serum free DMEM-F12 culture medium (Gibco) at a concentration of 10 μM DNCB, 0.1% ethanol.

Viability of cells after exposure to sensitizer was assayed using the Promega CellTiter 96 Non-Radioactive Cell Proliferation Assay, and cell toxicity to sensitizer was assayed using the Promega CytoTox 96 Non-Radioactive Cytotoxicity Assay (Supplementary Figure 2).

##### Flow cytometry

Cells were harvested by centrifugation and resuspended in 100 μl FACS buffer containing 10% FCS. One microgram per milliliters anti-HLA-A2 antibody BB7.2 (Parham and Brodsky, 1981) or anti-HLA-A, B, C antibody W6/32 (Barnstable *et al.*, 1978), or anti-MHC-2 (HB-145) (Shaw *et al.*, 1985) monoclonal antibodies were added and incubated for 30 min on ice. Excess antibodies were removed by centrifugation as previously described and cells were resuspended in 100 μl FACS buffer (1% BSA wt/vol, 1 mM EDTA in PBS) containing 1 μl per reaction FITC conjugated rabbit antimouse monoclonal antibodies and incubated for a further 30 min on ice. Cells were centrifuged as before and resuspended in 200 μl of 2% (wt/vol) formalin and incubated on ice for 30 min to fix the cells. Finally, cells were resuspended in FACS buffer prior to analysis using a Luminex Guava easyCyte flow cytometer (Merck) equipped with the relevant laser and filters to detect FITC fluorescence. Data were analyzed using the manufacturer software. MHC-I and -II Fluorescence Minus One Control were calculated by gating against cells incubated with secondary antibody alone.

##### Purification of HLA-I complexes and peptides

The snap frozen cell pellets (1 − 1.5 × 10${}^{8}$ cells) were briefly thawed prior to the addition of 8 ml lysis buffer (0.02 M Tris, 0.5% (wt/vol) IGEPAL, 0.25% (wt/vol) sodium deoxycholate, 0.15 mM NaCl, 1 mM EDTA, 0.2 mM iodoacetamide supplemented with EDTA-free protease inhibitor mix) and briefly mechanically dispersed using a pipette. Samples were left to solubilize for 30 min at 4°C. Homogenates were clarified for 10 min at 2000 × g, 4${}^{\circ }$C and then for a further 60 min at 13 500 × g, 4°C. Two milligrams of anti-MHC-I mouse monoclonal antibodies (W6/32) (Parham *et al.*, 1979) covalently conjugated to Protein A sepharose (Repligen) were added to the clarified supernatants and incubated with constant agitation for 2 h at 4°C. The captured HLA-I complex on the beads was washed sequentially with 10 column volumes of low (isotonic, 0.15 M NaCl) and high (hypertonic, 0.4 M NaCl) TBS washes prior to elution in 10% acetic acid and dried under vacuum.

The dried eluate was resuspended in 500 μl of 0.1% Trifluoroacetic acid (TFA)/1% acetonitrile (ACN) prior to injection into the Thermo UltiMate 3000 HPLC system using a Chromolith HighResolution RP-18 endcapped 100-4.6 HPLC column (Merck) to separate and collect the peptides for mass spectrometry analysis; 0.5 ml buffer A (0.1% TFA). Peptides were eluted with a linear gradient of 2%−30% buffer B (ACN and 0.1% TFA) were collected over 8 min. Fractions were pooled as odd and even fractions, lyophilized and then resuspended in 20 μl of 1% formic acid and split into 4 samples, 2 odd and 2 even, for mass spectrometry analysis.

##### Liquid chromatography with tandem mass spectrometry analysis of HLA-I peptides 

An HLA peptides were separated by an Ultimate 3000 RSLC nano system (Thermo Scientific) using a PepMap C18 EASY-Spray LC column, 2 μm particle size, 75 μm × 75 cm column (Thermo Scientific) in buffer A (0.1% formic acid) and coupled online to an Orbitrap Fusion Tribrid Mass Spectrometer (Thermo Fisher Scientific, United Kingdom) with a nanoelectrospray ion source. Peptides were eluted with a linear gradient of 3%−30% buffer B (ACN and 0.1% formic acid) at a flow rate of 300 nl/min over 110 min. Full scans were acquired in the Orbitrap analyzer using the Top Speed data dependent mode, preforming an MS scan every 3 s cycle, followed by higher-energy collision-induced dissociation (HCD) MS/MS scans. The MS spectra were acquired at resolution of 120 000 at 300 *m*/*z*, RF lens 60% and an automatic gain control ion target value of 4.0e5 for a maximum of 100 ms. The MS/MS resolution was 30 000 at 100 *m*/*z*. Higher-energy collisional dissociation (HCD) fragmentation was induced at an energy setting of 28 for peptides with a charge state of 2–4, whereas singly charged peptides were fragmented at an energy setting of 32 at lower priority. Fragments were analyzed in the Orbitrap at 30 000 resolution. Fragmented *m*/*z* values were dynamically excluded for 30 s.

##### Data analysis and code

Raw spectrum files were analyzed using Peaks Studio (Tran *et al.*, 2017; Zhang *et al.*, 2012) version X to the data processed to generate reduced charge state and deisotoped precursor and associated product ion peak lists which were searched against the Uniprot database (42 186 entries, 2017-05-18) and a contaminants list not excluding keratins in unspecific digest mode. Parent mass error tolerance was set a 5 ppm and fragment mass error tolerance at 0.03 Da. Variable modifications were set for N-term acetylation (42.01 Da), methionine oxidation (15.99 Da), carboxyamidomethylation (57.02 Da) of cysteine, and DNP modification (166.00 Da) of cysteine and lysine. A maximum of 3 variable modifications per peptide were set. The false discovery rate (FDR) was estimated with decoy-fusion database searches (Zhang *et al.*, 2012) and were filtered to 1% FDR.

Downstream analysis and data visualizations of the Peaks Studio identifications was performed in R using associated packages (Gehlenborg, 2019; Jessen, 2018; Pedersen, 2019; R Core Team, 2018; Wickham *et al.*, 2019).

The mass spectrometry proteomics data have been deposited to the ProteomeXchange Consortium via the PRIDE (Perez-Riverol *et al.*, 2019) partner repository with the dataset identifier PXD021373 and 10.6019/PXD021373.

##### ELISpot assays

Induction of ACD by DNCB in humans has been previously utilized as an immunostimulatory therapy for treatment of various skin conditions. Therefore, with ethical approval (REC reference number 16/LO/2176), we recruited a DNCB allergic volunteer (IMS1) through the dermatology clinic, University Hospital Southampton NHS Foundation Trust. Importantly, we could confirm that the volunteer had been sensitized to DNCB following skin exposure and had previously shown a positive patch test to DNCB following sensitization (Supplementary Table 1).

ELISpot assays were performed at least in duplicate. The protocol used was in accordance with manufacturer’s instructions (Mabtech AB ELISpot kit) with minor adjustments. Multiscreen-IP 96-well plates (Millipore) were coated with mouse anticytokine antibody as per manufacturer’s instructions using 2 × 10^5^ PBMCs. Peptide was added at a final concentration of 8μM. PHA was added to a final concentration of 4 μg/ml. Plates were left overnight (16 h) at 37°C with 5% CO_2_. Assay spots were enumerated using an automated ELISpot reader (AID, Germany). Peptide-specific reactivity was calculated by subtracting the counts from control wells, and the results expressed as spot-forming units (sfu) per million lymphocytes plated.

## RESULTS

Our previous studies included examination of the haptenation of the lysed HaCaT cell proteome that we had previously used to examine sensitizer haptenation of the proteome (Parkinson *et al.*, 2018), as well as DNCB haptenation of the living HaCaT cell line (Parkinson *et al.*, 2020). HaCaT cells are a spontaneously immortalized keratinocyte cell line (Boukamp *et al.*, 1988), and previous gene expression profiling of HaCaT cells indicated the presence of an HLA-A2 allele at the A locus (Lemaitre *et al.*, 2004). However, HLA typing (Supplementary Table 2) revealed HaCaTs to be homozygous for HLA-A*31:01, and the absence of HLA-A2 was subsequently confirmed via flow cytometry using HLA-A2-specific antibody BB7.2 (Parham and Brodsky, 1981) (Supplementary Figure 1). HLA-A*02:01 and HLA-A*31:01 are present in the U.K. population at frequencies of approximately 1 in 2, and 1 in 17, respectively (González-Galarza *et al.*, 2015). To maximize the chances of matching proteomic observations with recruited patients we created an HLA-A2 transfected HaCaT cell line (HaCaT HLA-A2). Flow cytometry analysis of the surface expression of both the wild-type HaCaT and HLA-A2 transfectant indicated expression of class I, but only low levels of class II HLA molecules (Supplementary Figure 1). This is consistent with HaCaTs having a keratinocyte phenotype, rather than that of professional antigen presenting cells. Consistent with previous observations of the toxicity of DNCB to cells (Chia *et al.*, 2010; Spriggs, 2017), we found cells remained viable after exposure to a concentration of 10 μM DNCB for 24 h, but that at higher concentrations toxicity was substantial (>10% of cells) (Supplementary Figure 2). Hence, we chose a 10 μM DNCB treatment for 24 h as our experimental sensitizer exposure.

To investigate the 3 models for how haptens mediate CTL recognition, HaCaT cells and HaCaT HLA-A2 cells were cultured either with or without 10 μM DNCB before harvesting and immunoaffinity purification of HLA-I peptides. The peptidome was subsequently characterized by liquid-chromatography-mass spectrometry proteomics and bioinformatic analysis.

###  

#### No Evidence for DNCB Induction of an Altered Peptidome

The class I HLA-peptide repertoire presented at the cell surface, the peptidome represents a snapshot of the internal proteome and antigen processing machinery. In conjunction with the intrinsic properties of each HLA allotype (Bailey *et al.*, 2015; Guo *et al.*, 1992; Zhang *et al.*, 1998), various hypotheses propose the relative importance of a number of factors influencing the peptidome, including: The abundance of source proteins from which the peptidome derives (Bassani-Sternberg *et al.*, 2015; Boulanger *et al.*, 2018; Dersh and Yewdell, 2016), the turnover rate of source proteins (Bassani-Sternberg *et al.*, 2015; Milner *et al.*, 2006), the synthesis rate of source proteins (Croft *et al.*, 2013; Yewdell *et al.*, 2003), the size of source proteins (Hoof *et al.*, 2012), the effects of cytokines on the immunoproteasome (Boulanger *et al.*, 2018; Chong *et al.*, 2018; Kesmir *et al.*, 2003), and the antigen processing machinery itself, namely TAP, tapasin, TABPR, and ERAP (Boyle *et al.*, 2013; Day *et al.*, 1995; Howarth *et al.*, 2004; Reeves and James, 2018). A unified understanding of the contribution of these factors to the peptidome does not exist, but by perturbing HaCaT cells with DNCB we sought to test whether DNCB could lead to an altered peptidome.

**Figure 1.**
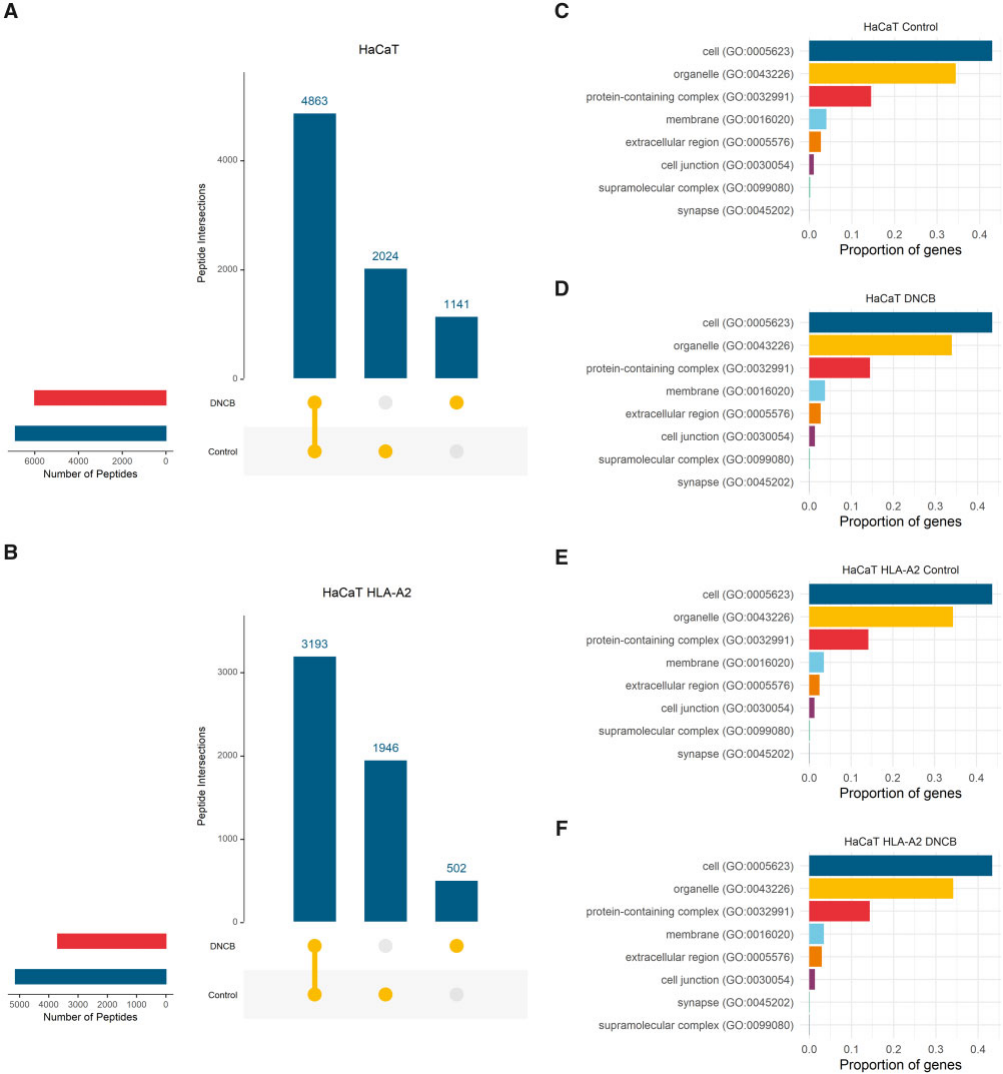
No evidence for altered peptidome by DNCB modulation of the proteome: A and B, Upset plots (Conway *et al.*, 2017) of intersections of distinct HLA-I peptides observed in 3 biological replicates of control and DNCB treated HaCaT and HaCaT HLA-A2 cells. The total number of distinct 8 − 15mer peptides observed for each condition are shown on the side bars. C and F, Cell component gene ontology classification of distinct genes from which peptidome originates using PANTHER (Mi *et al.*, 2019) from 3 biological replicates of control and DNCB treated HaCaT and HaCaT HLA-A2 cells.

From 3 biological replicates (150 × 10^6^ cells) a total of 8028 unique peptide sequences were identified for peptides between 8 and 15 amino acids in length (8 − 15mers) in DNCB treated or control HaCaT cells. More than half (4863; 61%) of peptides these were shared between both conditions (Figure 1A). Similar results were obtained for the 3 biological replicates of HaCaT HLA-A2. 5641 distinct 8 − 15mer peptides were identified across both conditions. Of these peptides 3193 (57%) were shared (Figure 1B). In both cell lines, DNCB exposure led to identification of fewer distinct peptides (Figs. 1A−B). There was no evidence of reduced cell quantity following this treatment. To further examine whether DNCB was perturbing the proteome, we undertook gene ontology classification of the peptidome source proteins using PANTHER (Mi *et al.*, 2019) and classified by cell compartment source expression. We found no evidence that DNCB treatment modified the cellular compartment proportions of proteins processed for presentation for either HaCaT or HaCaT HLA-A2 cells (Figs. 1C−F).

#### No Evidence for an Altered Peptidome by DNCB Modification of HLA Molecules

**Figure 2.**
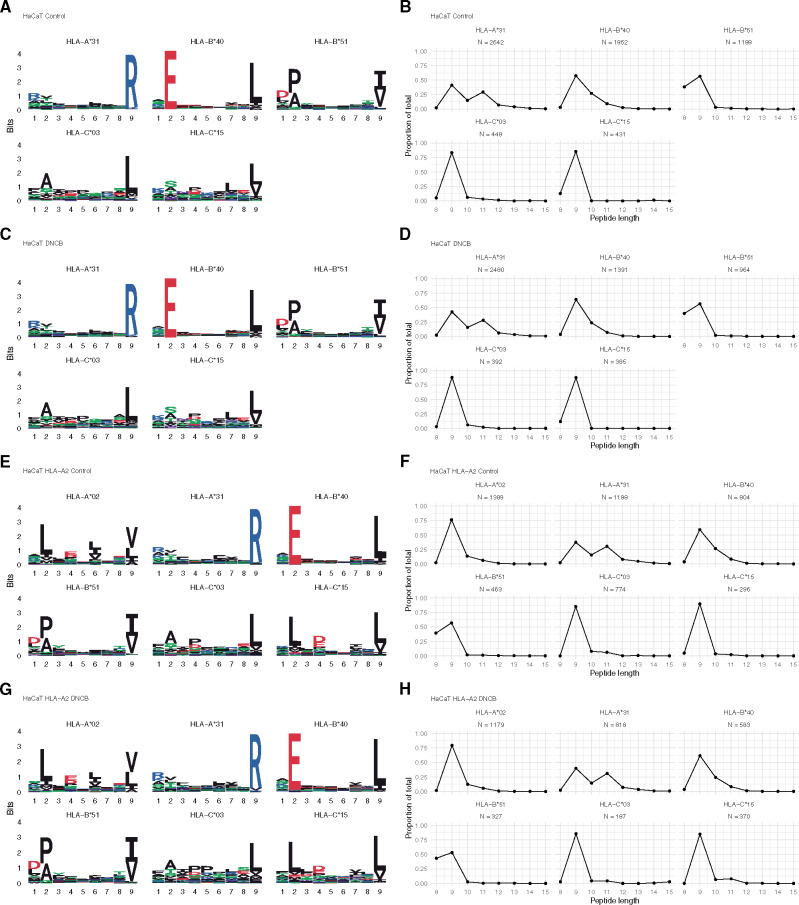
No evidence for an altered peptidome by DNCB modification of HLA molecules: A, C, E, G, HLA molecule 9-mer binding motifs ±DNCB treatment (Bassani-Sternberg and Gfeller, 2016; Gfeller *et al.*, 2018; Jessen, 2018). B, D, F, H, HLA bound peptide length distributions ±DNCB treatment (Bassani-Sternberg and Gfeller, 2016; Gfeller *et al.*, 2018).

Geometric and biochemical properties of the peptide binding groove of HLA-I molecules give rise to the peptide binding motif as denoted by the amino acid preference at each position along the peptide (Zhang *et al.*, 1998), and/or the peptide length preference (Guo *et al.*, 1992). To explore the possibility of DNCB modification of HLA molecules and subsequent CTL activation as seen with abacavir (Illing *et al.*, 2012), we examined whether exposure to DNCB changed the HLA-I binding motif or distribution of peptide length in the peptidome (Bassani-Sternberg and Gfeller, 2016; Gfeller *et al.*, 2018). We found that DNCB treatment had no effect on the HLA binding motifs (Figs. 2A, 2C, 2E, and 2G) or the length distributions of the peptides in the peptidome (Figs. 2B, 2D, 2F, and 2H).

#### Identification of DNCB Modified Peptides

**Figure 3.**
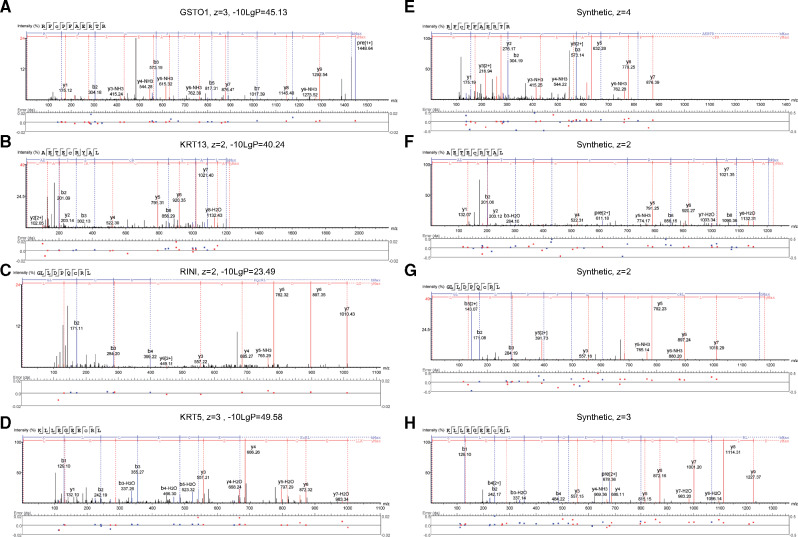
DNCB modified HLA peptide peptide-spectrum matches: A, B, C, D, Peptide-spectrum matches for RFC(+DNP)PFAERTR DNCB modified HLA-A31 peptide from glutathione-S-transferase omega-1 (Uniprot: P78417), AETEC(+DNP)RYAL DNCB modified HLA-B40 peptide from Keratin, type I cytoskeletal 13 (Uniprot: P13646), GLLDPQC(+DNP)RL DNCB modified HLA-A2 peptide from Ribonuclease inhibitor (Uniprot: P13489) and KLLEGEEC(+DNP)RL DNCB modified HLA-A2 peptide from Keratin, type II cytoskeletal 5 (Uniprot: P13647). E, F, G, H, Peptide-spectrum matches for RFC(+DNP)PFAERTR, AETEC(+DNP)RYAL, GLLDPQC(+DNP)RL, KLLEGEEC(+DNP)RL synthetic peptides.

To eliminate false positive identification of DNCB modified peptides, the DNCB treatment comprised 50:50 mixture of DNCB and deuterated DNCB-D_3_. DNCB modifications were confirmed by the assignment of the ion pairs corresponding to the addition of DNP (Δ = 166.0 Da) and corresponding DNCB-D${}_{3}$ modification 3 *m*/*z* units higher (Δ = 169.00 Da). For example, for a peptide ionized with a charge of +2, the deuterated peptide ion would be expected at 3/2 = 1.5 *m*/*z* units higher than the corresponding undeuterated peptide ion, as for example, in Supplementary Figure 3A. These identifications were then further confirmed by examination of the peptide-spectrum matches (Figure 3) and comparison with peptide-spectrum matches from synthetic peptides (Figs. 3E−H). We identified 4 DNCB haptenated peptides (Figure 3) from 3 HLA allotypes: (1) HLA-A*31:01 peptide deriving from glutathione-S transferase omega-1 (Uniprot: P78417) identified in 2 biological replicates (Figure 3A) with a DNP modification in the active site cysteine to which glutathione binds (Supplementary Figure 5). (2) HLA-B*40:01 peptide deriving from Keratin, type I cytoskeletal 13 (Uniprot: P13646) identified in 2 biological replicates (Figure 3B). (3) An HLA-A*02:01 peptide identified from Ribonuclease inhibitor (Uniprot: P13489) in 1 biological replicate (Supplementary Figure 6). (4) HLA-A*02:01 (Figure 3C). Peptide identified in 2 biological replicates from Keratin, type II cytoskeletal 5 (Uniprot: P13647), respectively (Figure 3D). All 4 proteins from which the identified presented peptides derive, were previously observed in the HaCaT proteome (Parkinson *et al.*, 2020), however, the DNP-Cys modification of Keratin 5 was the only modification observed in the earlier study.

**Figure 4.**
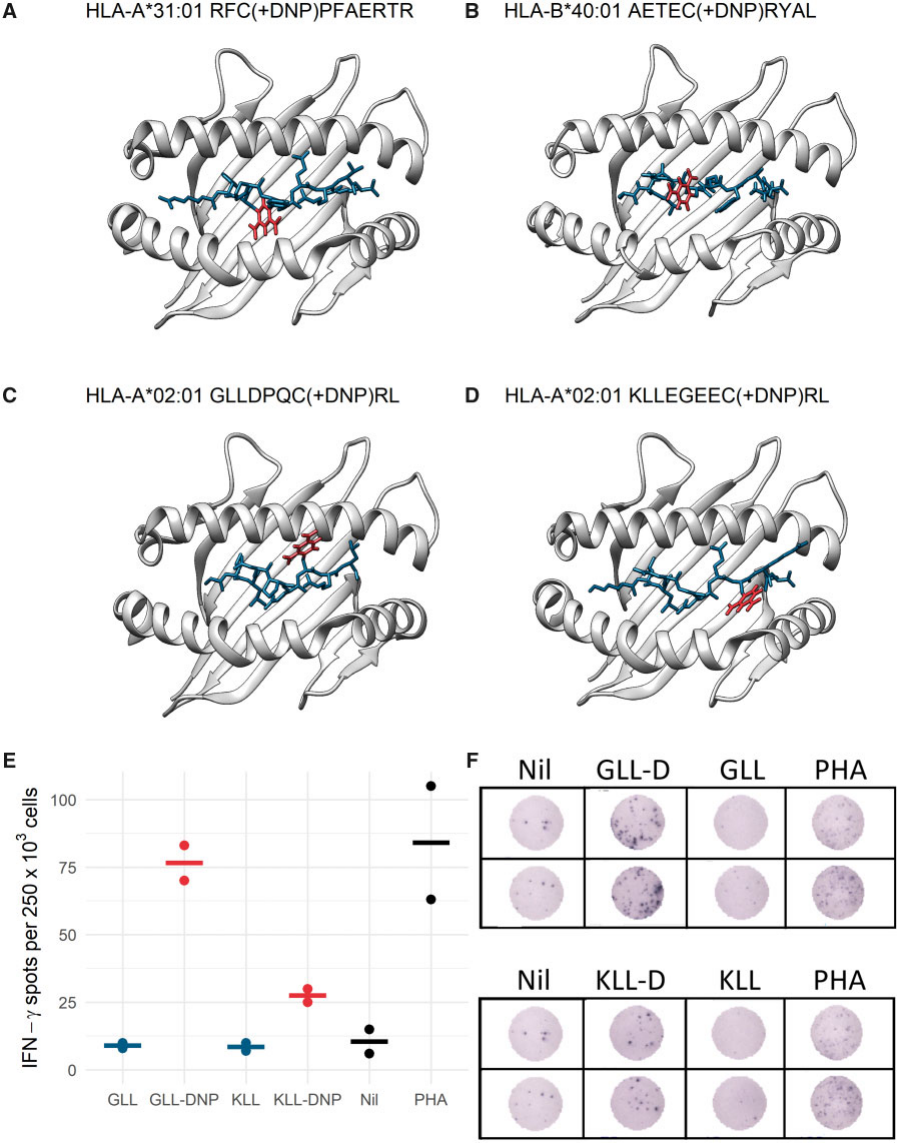
DNCB modified HLA peptides model structures and functional assay: A, B, C, D, Homology models of DNP modified peptides: Putative structures were created of the DNP modified peptides using USCF Chimera (Pettersen *et al.*, 2004; Shapovalov and Dunbrack, 2011) using PDB: 1IM3. E, F, Representative images from *ex vivo* IFN-γ ELISpot assay or enumerated spots after short term culture. Freshly isolated PBMC from an HLA-A*02:01 individual IMS1 were incubated with dinitrophenylated HLA-A*A02:01 GLL-D, KLL-D, or matched nondinitrophenylated peptides (GLL, KLL) before activation in an ELISpot assay with nil, peptides, or phytohemagglutinin.

To examine whether these modifications were likely to be visible to T-cell receptors on CTL, rather than buried between the peptide and HLA molecule, putative structures were created of the DNP modified peptides using USCF Chimera (Pettersen *et al.*, 2004): HLA-A*31:01 and DNP modified peptide from glutathione-S transferase (RF(C-DNP)PFAERTR) (Figure 4A); HLA-B*40:01 with superposition of the identified DNP modified peptide AETE(C-DNP)RYAL from Keratin type I, cytoskeletal 13 (Figure 4B); HLA-A*02:01 and DNP modified peptide GLLDPQ(C-DNP)RL from Ribonulease inhibitor (Figure 4C); HLA-A*02:01 and DNP modified peptide KLLEGEE(C-DNP)RL from Keratin type II, cytoskeletal 5 (Figure 4D). These models indicate that the position of the peptide modification within each structure are likely to be solvent exposed and oriented toward CTL interaction, however, no crystal structures exist for these peptides and the dynamic nature of proteins means that other peptide configurations are also possible. For an HLA-A2 positive patient previously sensitized to DNCB, we observed CTL responses to the dinitrophenylated form of the HLA-A2 peptides, but not to the nondinitrophenylated HLA-A2 peptides (Figs. 4E and 4F).

## DISCUSSION

In this study, we sought to examine the effects of DNCB on the peptidome of keratinocytes and the events leading to skin sensitization. With the knowledge of the level and specificity of DNCB haptenation in HaCaT cells (Parkinson *et al.*, 2020), we investigated 3 nonmutally exclusive models for hapten modulation of the peptidome: Presentation of haptenated peptides; hapten modification of HLA leading to an altered HLA-peptide repertoire; or a hapten altered proteome leading to an altered HLA-peptide repertoire. Of these 3 models, we found evidence only for the presentation of haptenated peptides. We found no evidence of changes in the lengths, or amino acid composition of the presented peptides, or in the source proteins from which the peptides derive. Additionally, we found a preference for cysteine DNP modification of haptenated peptides, and evidence of DNCB in the cellular detoxification pathway.

These findings are consistent with the observation that CD8+ CTLs are the primary mediators of the immune response to haptens (Martin *et al.*, 1993b; Vocanson *et al.*, 2005, 2009; Weltzien *et al.*, 1996). Synthetic dinitrophenylated peptides have been shown to generate HLA and peptide-specific CTL from donors who had not previously been exposed to DNCB (Gagnon *et al.*, 2003), with similar observations using synthetic trinitrophenylated peptides in mice (Ortmann *et al.*, 1992; Von Bonin *et al.*, 1992). However, it is possible whilst the peptides are HLA restricted by binding motif, they maybe reactive to multiple CTLs. Where CTLs were generated following priming with trinitrophenylated peptides, multiple CTLs were reactive to H2-K^b^ molecules bearing different haptenated peptides (Franco *et al.*, 1999; Martin *et al.*, 1992). Our observation that dinitrophenylation occurs away from peptide anchor positions corresponds with observations of trinitrophenylated peptides bound to H2-K^b^ (Martin *et al.*, 1992, 1993a). Moreover, our models of dinitrophenylated peptide HLA structures suggest that DNP is exposed to the CTL analogous to observed structures of class I MHC bound glycopeptides recognized by glycan-specific CTLs (Glithero *et al.*, 1999; Speir *et al.*, 1999).

We observed a peptide-specific CTL response for 2 HLA-A2 dinitrophenylated peptides in a patient previously sensitized to DNCB, but we were unable to confirm whether these peptides could be recognized by an unsensitized patient, or whether these peptides haptenated with different sensitizers would also stimulate the CTLs in our patient, as previously reported (Gagnon *et al.*, 2006).

Our observations cannot specifically confirm that haptenation has taken place within the cell or at the cell surface. Our observations cannot specifically confirm that haptenation has taken place within the cell or at the cell surface. The unmodified form of peptide KLLEGEECRL from Keratin type II, cytoskeletal 5 has been previously reported with IEDB epitope ID 774189 (Vita *et al.*, 2019) indicating the availability of the cysteine for extracellular modification. However, the dinitrophenylated cysteines that we observe on HLA peptides are from cytosolic proteins with free cysteines within the intracellular environment. Cysteines on keratins, including KRT5, have previously been identified as a target for haptenation (Bauer *et al.*, 2011; Simonsson *et al.*, 2011). Given the ubiquity of keratins in the epidermis, a role for them in ACD would be unsurprising. Our identification of dinitrophenylated peptides from 2 keratins, and the observation that only the dinitrophenylated peptide could stimulate CTL (Supplementary Figure 7) supports the hypothesis that haptenation of keratins plays a key role in induction and elicitation of ACD (Aleksic *et al.*, 2008). Ribonuclease inhibitor (RINI) consists of 15 leucine rich repeats containing 32 cysteines, the majority of which are in a reduced form (Dickson *et al.*, 2005; Lee *et al.*, 1988) (Supplementary Figure 6). Oxidation of free cysteines using 2,4-dinitrothiocyanatebenzene (DNTB) has been shown to inactivate RINI and trigger intracellular degradation (Blázquez *et al.*, 1996; Fominaya and Hofsteenge, 1992). The sensitivity of RINI to oxidation has led to suggestions that RINI may have a role in protecting cells against oxidative stress (Cui *et al.*, 2003). Binding of DNCB to the cysteine of glutathione mediated by glutathione-S transferases (GST) is the key mechanism by which the DNCB is removed from cells (Townsend *et al.*, 2003). Our observation of a dinitrophenylated peptide arising from the active site of cytosolic glutathione-S transferase-ω (GSTO1) provides further evidence that our observed peptides were processed internally, and importantly indicate that DNCB is a substrate for GSTO1. Depletion of glutathione by DNCB has be linked to the triggering of the nuclear factor E2-related factor 2 (Nrf2) pathway that regulates the expression of antioxidant proteins which include GSTs (Jacquoilleot *et al.*, 2015). Our observations imply that DNCB has the potential to add to oxidative stress not only by depleting glutathione, but also by disrupting the function of GSTs.

In conclusion, our findings identify haptenated HLA peptides presented by HLA molecules. The motif of the peptides matched their corresponding HLA molecules, implying HLA-restriction. A limitation to this work is that we were unable to test the HLA-restriction of these peptides and CTL specificity in patients. In experimental models where reduced cysteines are absent DNCB haptenates lysines preferentially (Lepoittevin, 2006; Parkinson *et al.*, 2014). In contrast, we observed only cysteine modified peptides and it is notable that these cysteines would all be reduced in the intracellular environment, indicating that proteins with free cysteines may have an important role in the generation of hapten responsive CD8+ CTLs. Finally, the identification of a dinitrophenylated peptide deriving from the active site of a glutathione-S-transferase suggests an additional mechanism by which DNCB increases oxidative stress leading to cell death.

## SUPPLEMENTARY DATA

Supplementary data are available at *Toxicological Sciences* online.

## DECLARATION OF CONFLICTING INTERESTS

The authors declared no potential conflicts of interest with respect to the research, authorship, and/or publication of this article.

## Supplementary Material

kfaa184_Supplementary_Data
