# Supplementary material for: Characterization of the Class I MHC Peptidome Resulting From DNCB Exposure of HaCaT Cells
Source: Toxicol Sci. 2020 Dec 29;180(1):136–47. doi: 10.1093/toxsci/kfaa184 (PMC7916740; doi:10.1093/toxsci/kfaa184)
Supplement: kfaa184_Supplementary_Data [file kfaa184_supplementary_data.docx]

# Supplementary Material

# Characterization of the class I MHC peptidome resulting from DNCB exposure of HaCaT cells

Alistair Bailey^1,2^, Ben Nicholas^1,2^, Rachel Darley^2^, Erika Parkinson^1^, Ying Teo^3^, Maja Aleksic^4^, Gavin Maxwell^4^, Tim Elliott^2^, Michael Ardern-Jones^3^, Paul Skipp^1^

^1^Centre for Proteomic Research, Biological Sciences and Institute for Life Sciences, Building 85, University of Southampton, SO17 1BJ, UK

^2^Centre for Cancer Immunology and Institute for Life Sciences, Faculty of Medicine, University of Southampton, SO16 6YD, UK

^3^Clinical and Experimental Sciences, Sir Henry Wellcome Laboratories, Faculty of Medicine, University of Southampton, SO16 6YD, Southampton, UK

^4^Safety & Environmental Assurance Centre, Unilever, Colworth Science Park, Sharnbrook, MK44 1LQ, UK

### Correspondence to:

Dr Paul J. Skipp

Centre for Proteomic Research
B85, Life Sciences Building
University of Southampton
University Road
Highfield
Southampton, Hants.
SO17 1BJ

Tel No: 023 80594204

email: [pjss@soton.ac.uk](mailto:pjss@soton.ac.uk)

## Supplementary Material

### Supplementary Table 1: Patient information

Induction of allergic contact dermatitis by DNCB in humans has been previously utilized as an immunostimulatory therapy for treatment of various skin conditions. Therefore, with ethical approval (REC reference number 16/LO/2176), we recruited a DNCB allergic volunteer (IMS1) through the dermatology clinic, University Hospital Southampton NHS Foundation Trust. Importantly, we could confirm that the volunteer had been sensitised to DNCB following skin exposure and had previously shown a positive patch test to DNCB following sensitisation.


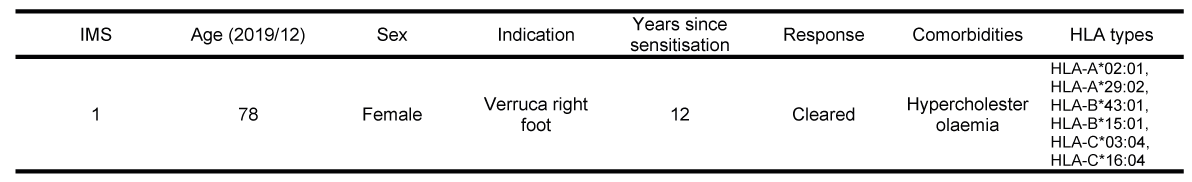


### Supplementary Table 2 : HLA type of HaCaT cells

HLA typing was performed by Next Generation Sequencing by the NHS Blood and Transplant Histocompatibility and Immunogenetics Laboratory, Colindale, UK.


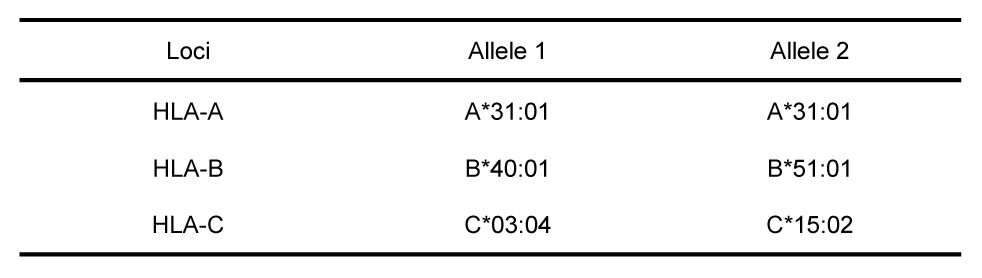


### Supplementary Figure 1

Cells were harvested by centrifugation and re-suspended in 100$\mu$l FACS buffer containing 10% FCS. 1$\mu$g/ml anti-HLA-A2 antibody BB7.2 (Parham and Brodsky, [1981](#ref-parham1981)) or anti-HLA-A, B, C antibody W6/32 (Barnstable *et al.*, [1978](#ref-barnstable1978)) or anti-MHC-2 (HB-145) (Shaw *et al.*, [1985](#ref-shaw1985)) monoclonal antibodies were added and incubated for 30 min on ice. Excess antibodies were removed by centrifugation as previously described and cells were re-suspended in 100$\mu$l FACS buffer containing 1$\mu$l per reaction FITC conjugated rabbit anti-mouse monoclonal antibodies and incubated for a further 30 min on ice. Cells were centrifuged as before and re-suspended in 200$\mu$l of 2% (w/v) formalin and incubated on ice for 30 min to fix the cells. Finally, cells were re-suspended in FACS buffer prior to analysis using a Luminex Guava easyCyte flow cytometer equipped with the relevant laser and filters to detect FITC fluorescence. Data were analysed using GUAVA software. MHC-I and –II Fluorescence Minus One Control were calculated by gating against cells incubated with secondary antibody alone.


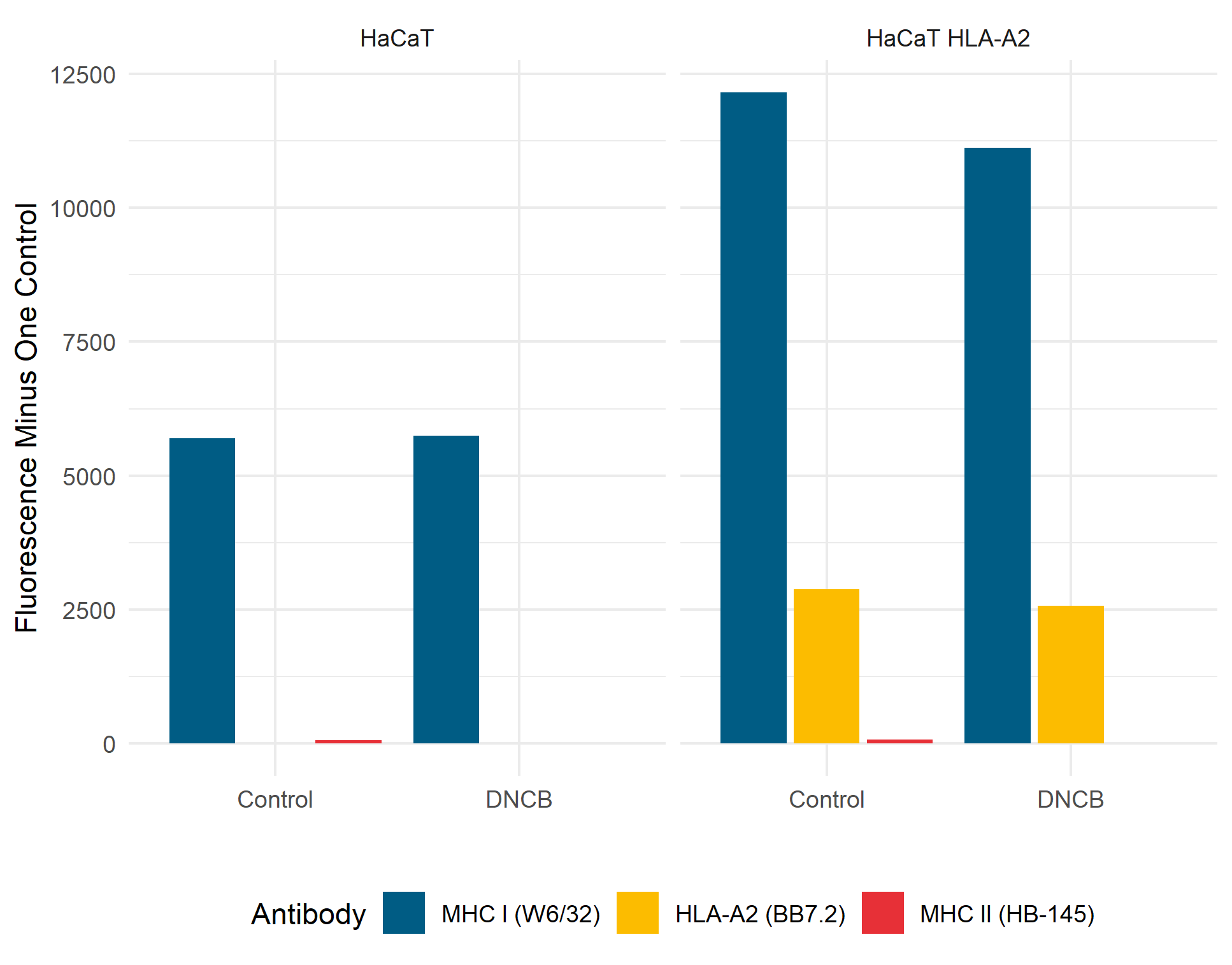


Supplementary Figure 1: Analysis of HaCaT surfcace HLA expression: cell HLA phenotype was confirmed by staining with anti-HLA-A2 antibody BB7.2(Parham and Brodsky, [1981](#ref-parham1981)) or anti-HLA-A, B, C antibody W6/32 (Barnstable et al., [1978](#ref-barnstable1978)) or anti-MHC-2 (HB-145) (Shaw et al., [1985](#ref-shaw1985)) followed by goat anti-mouse conjugated with FITC (Sigma-Aldrich) and analysed by flow cytometry (Luminex Guava easyCyte).

### Supplementary Figure 2


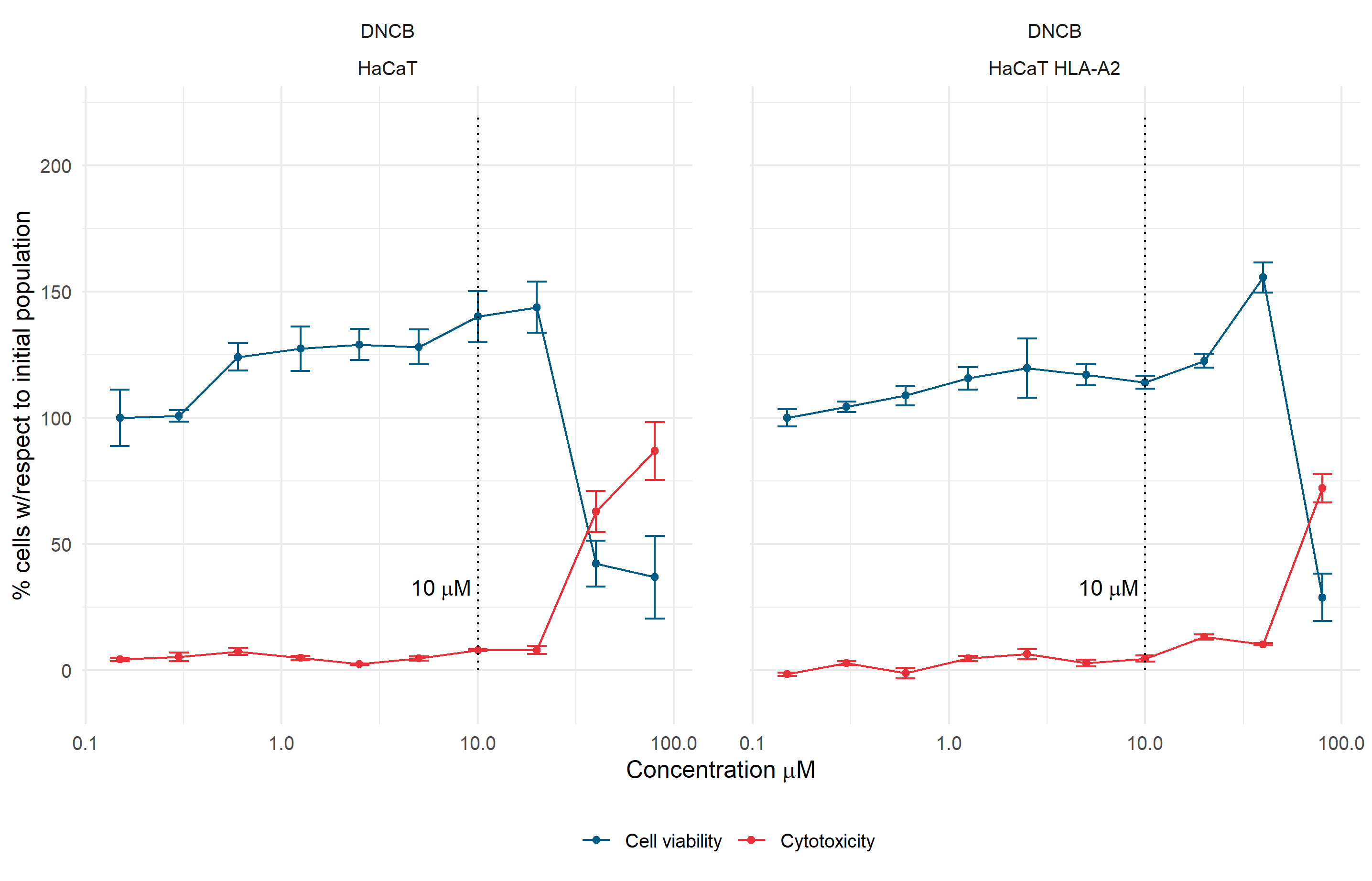


Supplementary Figure 2: Cell viability/Cytotoxicity Assays: Viability of cells after exposure to sensitiser was assayed using the [Promega CellTiter 96 Non-Radioactive Cell Proliferation Assay](file:///C:\Users\44793\Documents\hacat-peptidome-paper-2020\R\www.promega.co.uk), a colorimetric method for determining the number of viable cells. Cell toxicity to sensitiser was assayed using the [Promega CytoTox 96 Non-Radioactive Cytotoxicity Assay](file:///C:\Users\44793\Documents\hacat-peptidome-paper-2020\R\www.promega.co.uk), a colorimetric cytotoxicity assay that quantitatively measures lactate dehydrogenase (LDH) release upon cell lysis.

### Supplementary Figure 3


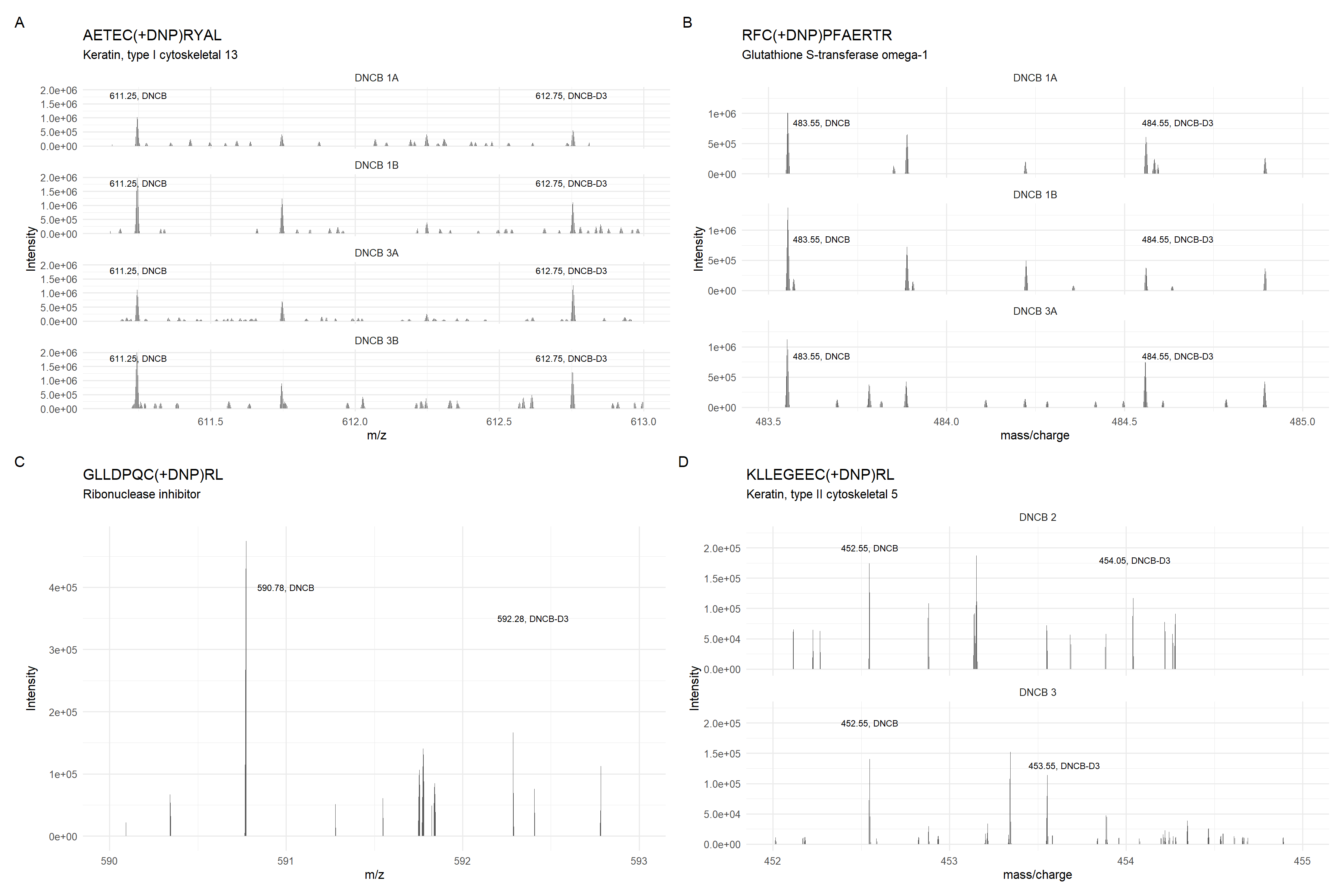


Supplementary Figure 3: MS spectra of DNP modified HLA peptides: Cells were treated with 50:50 mixture of DNCB:DNCB-D_3. DNCB treatment comprised of 50:50 mixture of DNCB and deuterated DNCB-D${}_{3}$. DNCB modifications were confirmed by the assignment of the ion pairs corresponding to the addition of DNP ($\Delta$ = 166.0 Da) and corresponding DNCB-D${}_{3}$ modification three m/z units higher ($\Delta$ = 169.00 Da). For a doubly charged peptide the m/z increase is 1.5 and for a triply charged peptide the m/z increase is 1. (A) Doubly charged AETEC(+DNP)RYAL DNP modified HLA-B40 peptide from Keratin, type I cytoskeletal 13 (Uniprot:P13646). (B) Triply charged RFC(+DNP)PFAERTR DNP modified HLA-A31 peptide from Glutathione S-transferase omega-1 (Uniprot:P78417). (C) Doubly charged GLLDPQC(+DNP)RL DNP modified HLA-A2 peptide from Ribonuclease inhibitor (Uniprot:P13489). (D) Doubly charged KLLEGEEC(+DNP)RL DNP modified HLA-A2 peptide from Keratin, type II cytoskeletal 5 (Uniprot:P13647).

### Supplementary Figure 4


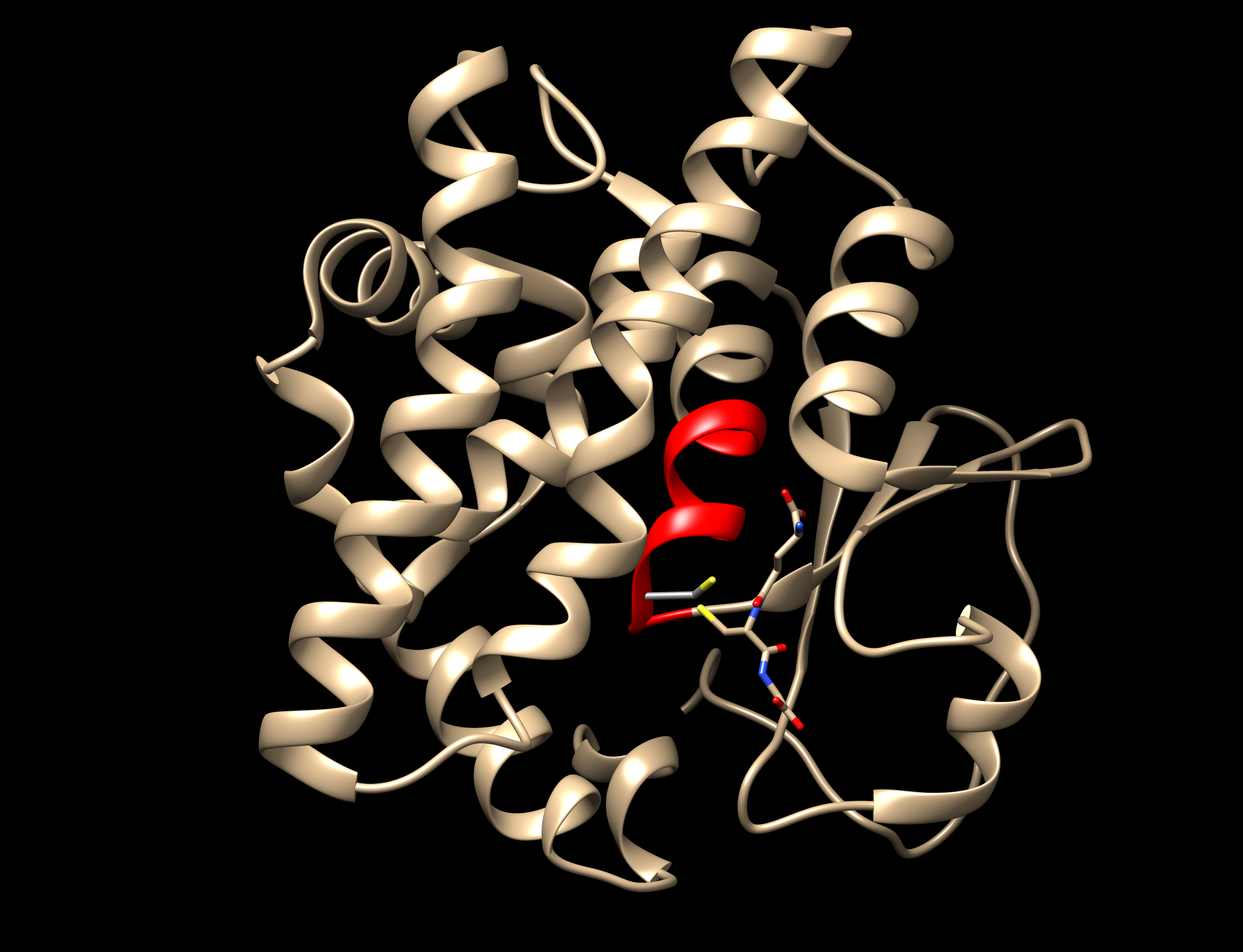


Supplementary Figure 4: Glutathione S-transferase omega-1 structure (Uniprot:P78417, PDB:5YVN), the peptide region is coloured red with the side chain shown for the modified free cysteine

### Supplementary Figure 5


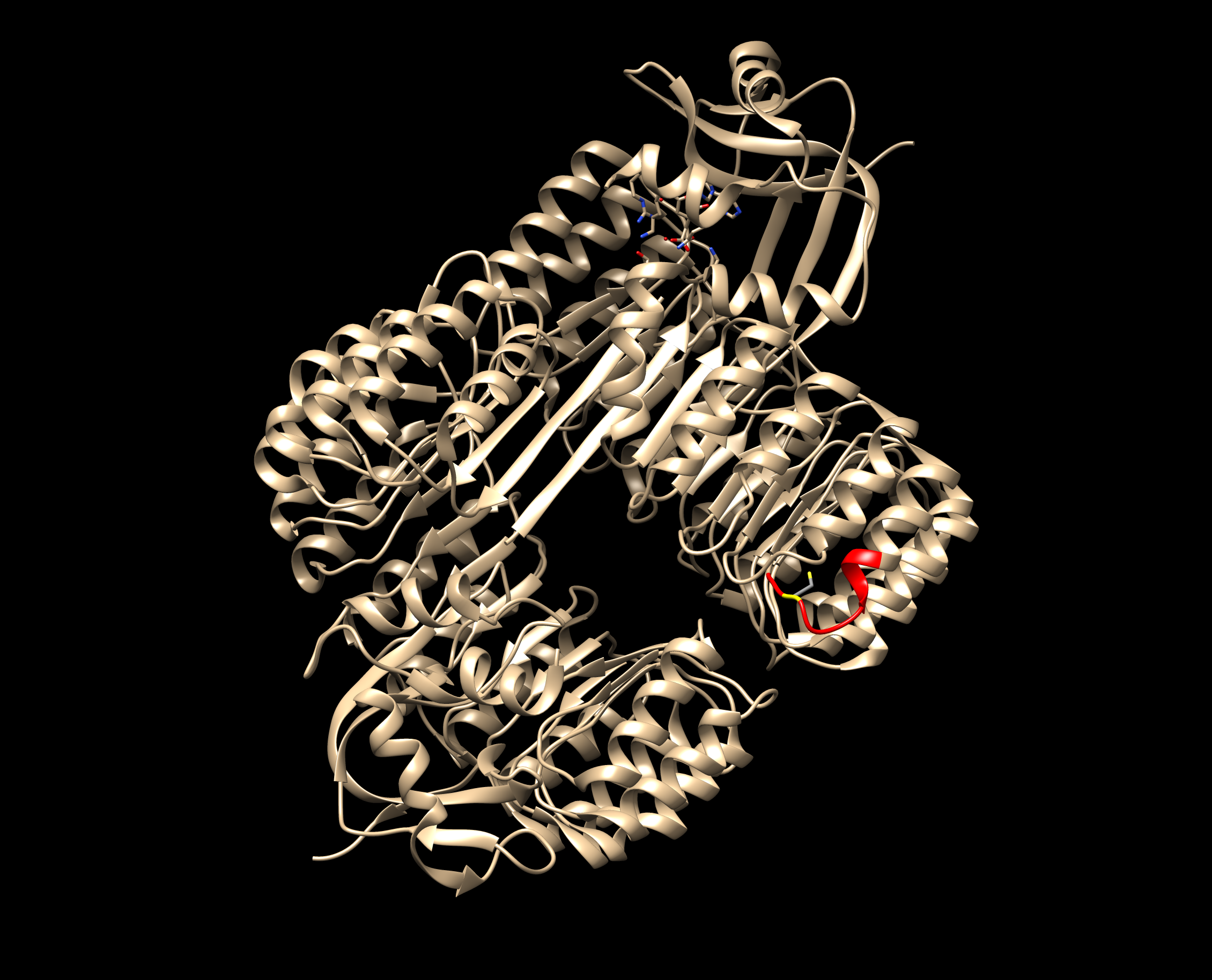


Supplementary Figure 5: Ribonuclease inhibitor structure (Uniprot:P13489, PDB:1A4Y), the peptide region is coloured red with the side chain shown for the modified free cysteine

### Supplementary References:

Barnstable, C.J. *et al.* (1978) Production of monoclonal antibodies to group a erythrocytes, hla and other human cell surface antigens-new tools for genetic analysis. *Cell*, **14**, 9–20.

Parham, P. and Brodsky, F.M. (1981) Partial purification and some properties of bb7. 2 a cytotoxic monoclonal antibody with specificity for hla-a2 and a variant of hla-a28. *Human immunology*, **3**, 277–299.

Shaw, S. *et al.* (1985) Specificity of monoclonal antibodies directed against human and murine class ii histocompatibility antigens as analyzed by binding to hla-deletion mutant cell lines. *Human immunology*, **12**, 191–211.
